# Non-motor symptoms in essential tremor, akinetic rigid and tremor-dominant subtypes of Parkinson’s disease

**DOI:** 10.1371/journal.pone.0245918

**Published:** 2021-01-27

**Authors:** Ali S. Shalash, Eman Hamid, Hanan Elrassas, Eshak I. Bahbah, Alia H. Mansour, Hadeer Mohamed, Mahmoud Elbalkimy

**Affiliations:** 1 Faculty of Medicine, Department of Neurology, Ain Shams University, Cairo, Egypt; 2 Okasha Institute of Psychiatry, Ain Shams University, Cairo, Egypt; 3 Faculty of Medicine, Al-Azhar University, Damietta, Egypt; National Institue on Drug Abuse, UNITED STATES

## Abstract

**Objectives:**

To compare non-motor symptoms (NMSs) among patients with essential tremor (ET), Parkinson’s disease (PD) subtypes (akinetic-rigid type (ART) and tremor-dominant type (TDT)), and healthy controls.

**Patients and methods:**

This retrospective study included 129 participants, 72 PD (33 PD-ART, 33 PD-TDT, and 6 Mixed), 29 ET patients, and 28 controls. PD patients were assessed by the unified Parkinson’s disease rating scale (UPDRS), Hoehn, and Yahr scale (H&Y), while ET patients were evaluated by the Fahn Tolosa Marin Tremor Rating Scale. All subjects were evaluated by non-motor symptoms scale (NMSS) for NMSs and Beck depression inventory (BDI) for depression.

**Results:**

PD subtypes groups, ET, and controls were age and gender-matched. Compared to controls, all PD, PD subtypes, and ET showed significantly worse most of NMSs (p<0.001) and depression. Compared to ET, all PD and PD-ART had significantly worse gastrointestinal (p = 0.002), urinary symptoms (p = 0.001, p = 0.003) and depression (p = 0.002) and PD-TDT worse depression, while ET patients showed worse memory/attention than PD subtypes. Total NMSS of ET is highly correlated to depression and moderately to tremor severity and age of onset, while total of NMSS is highly correlated to depression, disease severity, and disability.

**Conclusion:**

The current study demonstrated several comparable domains of NMSs of PD subtypes and ET, except worse gastrointestinal and urinary symptoms among PD-ART. Identifying different NMSs profiles is important for predicting, better assessing, and tailoring management of ET and PD subtypes.

## Introduction

Essential tremor (ET) and Parkinson’s disease (PD) are common tremor disorders in adults with increased prevalence with age. ET and PD affect about 4.6% [1] and 1% of populations older than 65 years [2] respectively and share overlapping clinical motor features, resulting in occasional difficulty to differentiate between them [3]. Both diseases were described mainly as motor disorders with different phenotypes, followed by recognition of associated nonmotor symptoms (NMSs). Currently, NMSs are considered an integral part in both disorders that significantly impact patients’ quality of life, demonstrating the importance of their assessment in patients’s management [3–5].

The postural and kinetic tremors of the upper limbs are the main symptoms of ET. However, other motor symptoms were reported, including gait ataxia, postural instability, disturbances of the eye movement, resting tremor, rigidity, and mild bradykinesia. Several NMSs are progressively being recognized with ET, including cognitive decline, depression, anxiety, pain, hearing impairment, and sleep problems [5,6].

Owing to the heterogeneous nature of PD, it can be classified into different subtypes; patients with predominant akinesia/rigidity that is an akinetic-rigid type (ART) and patients with a tremor-dominant type (TDT) [7]. In addition to motor differences, both subtypes showed variability of NMSs that might imply different pathophysiologies. For example, PD-ART is associated with greater cognitive impairment and more rapid progression than those with TDT-PD [8]. However, few previous studies reported inconsistent differences of associated NMSs between PD subtypes; therefore, further studies are warranted. On the other hand, the link between PD and ET has been suggested by previous clinical, imaging, and pathological studies [3,9]. Nevertheless, several issues of the ET- PD relationship are still controversial [9,10]. Investigating the differences and similarities of multiple aspects, including NMSs, between both disorders might help in clinical differentiation and understanding the relationship and underlying mechanisms of both disorders.

Previous studies of variable methodology and population compared NMSs among patients with PD, PD-TDT, and ET demonstrating a remarkable overlap [9]. However, the exact difference between PD and ET in terms of NMSs profile is still debatable. Kwon et al. [11], and Lageman et al. [12] reported that patients with PD had more NMSs compared to those with ET. On the contrary, Giorelli et al. [13] demonstrated a non-significant difference between patients with PD and ET in the total number of NMSs. Moreover, investigating NMSs profiles that distinguish between ET, PD-TDT, and PD-ART were not fully addressed. Hence, we aimed to explore differences of depression and NMSs between ET and PD phenotypes (PD-TDT and PD- ART) and their correlates.

## Methods

### Patients and study design

This retrospective study included 129 Egyptian adults from both genders; 72 patients with PD, 29 patients with a diagnosis of ET, and 28 healthy controls. Patients were recruited from the outpatient clinic at Ain Shams University Hospitals (Cairo, Egypt). Excluded subjects were patients with ET plus, atypical or acquired parkinsonism, patients with acquired, physiological, dystonic, or other types of tremor, and patients with a comorbid chronic illness that might affect NMSs. The matched controls were recruited from healthy volunteers, and persons accompanied patients with other diagnoses and were evaluated to exclude ET, PD, or the presence of a family history of tremor. All subjects were non-alcoholics according to cultural norms. All subjects were comprehensively evaluated as a part of previous studies and data registry of Movement disorders outpatient clinic. The Ethical Committee at the Faculty of Medicine, Ain Shams University, approved the study that is consistent with the ethical standards of the Declaration of Helsinki. Informed written consent was provided from all subjects.

### PD criteria

Patients with PD were diagnosed according to the British Parkinson’s disease Society Brain Bank criteria [14] and assessed by the unified Parkinson’s disease rating scale (UPDRS) and its subscores, Hoehn and Yahr scale (H&Y) for disease staging, and Schwab and England scale (S&E) for daily functioning, in medication "Off" and "On" states. Patients with PD were classified into tremor dominant type (PD-TDT) and akinetic rigid type (PD-ART), according to the ratio of the tremor score (sum of UPDRS Part III items 20 and 21, divided by 4) to the akinetic-rigid score (sum of UPDRS Part III items 22–27 and 31, divided by 15). A ratio ≥1 implies a tremor‐dominant type (TDT); a ratio of ≤0.80 implies akinetic‐rigid type (ART), while a ratio between 0.80 and 1.0 indicates a mixed type [7].

### ET criteria

Patients with ET were diagnosed according to International Parkinsonism and Movement Disorder Society (MDS) diagnostic criteria [15] and evaluated using the Fahn Tolosa Marin Tremor Rating Scale (FTMRS) for tremor severity [16].

### Non-motor symptoms

All subjects were evaluated using comprehensive history and neurological examination, Beck depression inventory (BDI) for depression [17], and non-motor symptoms scale (NMSS) for evaluating NMSs [18]. We used the available Arabic-validated version of BDI [19]. NMSS, is a 30 items questionnaire and composed of nine domains; cardiovascular (CVS), sleep/fatigue, mood/cognition, perceptual problems/hallucination, memory/attention, gastrointestinal tract (GIT), urinary, sexual, and miscellaneous symptoms. The frequency and severity of each NMS item were calculated, and the summary index for each domain was estimated (the sum of included items divided by the maximum possible score then multiplied by 100) to allow crude comparisons between the severity of different domains. the scale (English version) was demonstrated by expert through patients’ interview.

### Statistical analysis

The collected data were coded and analyzed using the Statistical Package for Social Science (SPSS version 22). Categorical data were presented as frequency and percentage and compared using the Chi-Square test, while continuous data were presented as mean and standard deviation (± SD) and compared using one-way ANOVA, accompanied by Tukey’s post hoc test. Multiple analyses were corrected with the Bonferroni method. Multiple linear regression analysis was performed. A p-value <0.05 was considered statistically significant. We performed two models; 1) unadjusted model and 2) adjusted model to age and BDI.

## Results

### Clinical and demographic characteristics of the study population

Seventy-two patients with PD (33 PD-ART, 33 PD-TDT, and 6 Mixed), 29 patients with ET, and 28 control individuals from both genders were enrolled in this retrospective study. The mean ages of patients with PD (all patients), ET, and controls were 53.02±10.05 years, 46.14±17.66 years, and 45.05 ± 16.71 years, respectively. The mean age of all patients with PD was comparable to those with ET (p = 0.60) and significantly higher than the mean age control group (p = 0.026). No significant difference between the study groups in terms of gender. Expectedly, the duration of the disease of ET patients (10.69±7.83 years) was significantly longer than patients with PD (5.01±3.87 years) and PD subtypes (p<0.001). No participants reported taking medications such as antidepressants that might affect NMSs, with only four patients were receiving propranolol to ameliorate tremor.

Mean ages of PD-TDT and ART subtypes were matched for ET (p = 0.207 and 0.457, respectively) and control groups (p = 0.113 and 0.291, respectively). Furthermore, the mean ages of ET and control groups were matched (p = 0.998). There was no significant difference between both groups PD-TDT and PD-ART, in terms of age, gender, age of onset, duration of disease, and motor functions (Table 1).

**Table 1:** Demographic and clinical characteristics of the study population.

	Total PD Mean (SD)	PD-TDT	PD-ART	ET Mean (SD)	Control Mean (SD)
**Number**	72	33	33	29	28
**Gender**					
** Male, *n* (%)**	38 (52.7%)	17 (51.5%)	18 (54.5%)	21 (72.4%)	15 (53.6%)
** Female, *n* (%)**	34 (47.3%)	16 (48.5%)	15 (45.5%)	8 (27.6%)	13 (46.4%)
**Age (years)**	53.02 (10.05) *	53.63 (8.81)	51.96 (11.29)	46.14 (17.66)	45.05 (16.71)
**Duration of illness (years)**	5.01 (3.87) †	5.5 (3.39) †	4.43 (4.26) †	10.69 (7.83)	-
**Age of onset (years)**	48.02 (10.95) †	48.19 (9.65) †	47.5 (12.24) †	35.37 (16.47)	-
**FTMRS**				42.51 (13.84)	
**H&Y Off**	2.77 (1.05)	2.60 (0.95)	2.91 (1.20)	-	-
**H&Y On**	1.09 (0.83)	1.03 (0.82)	1.21 (0.88)	-	-
**S&E Off**	54.92 (21.96)	59.68 (21.62)	50.30 (23.24)	-	-
**S&E On**	85.35 (14.32)	86.25 (12.88)	83.33 (16.32)	-	-
**UPDRS I**	3.92 (2.60)	3.75 (2.51)	4.12 (2.87)	-	-
**UPDRS II off**	18.44 (10.46)	17.93 (10.51)	18.21 (10.52)	-	-
**UPDRS II on**	6.10 (6.47)	5.45 (5.53)	6.09 (7.29)	-	-
**UPDRS III off**	38.57 (19.31)	40.24 (21.02)	37.03 (18.55)	-	-
**UPDRS III on**	14.68 (12.73)	14.54 (13.11)	14.87 (13)	-	-

PD, Parkinson’s Disease; ET, essential tremor; SD, standard deviation; FTMRS, Fahn–Tolosa–Martin Tremor Rating Scale; H&Y, Hoehn and Yahr Scale; S&E, Schwab and England Scale, UPDRS, Unified Parkinson’s Disease Rating Scale; TDT, tremor dominant type; ART, akinetic rigid type.

*P<0.05 compared to control.

^†^P<0.05 compared to ET group.

P-value was adjusted using Bonferroni method.

### Comparison of non-motor symptoms of whole PD and ET groups

**Fig 1:**
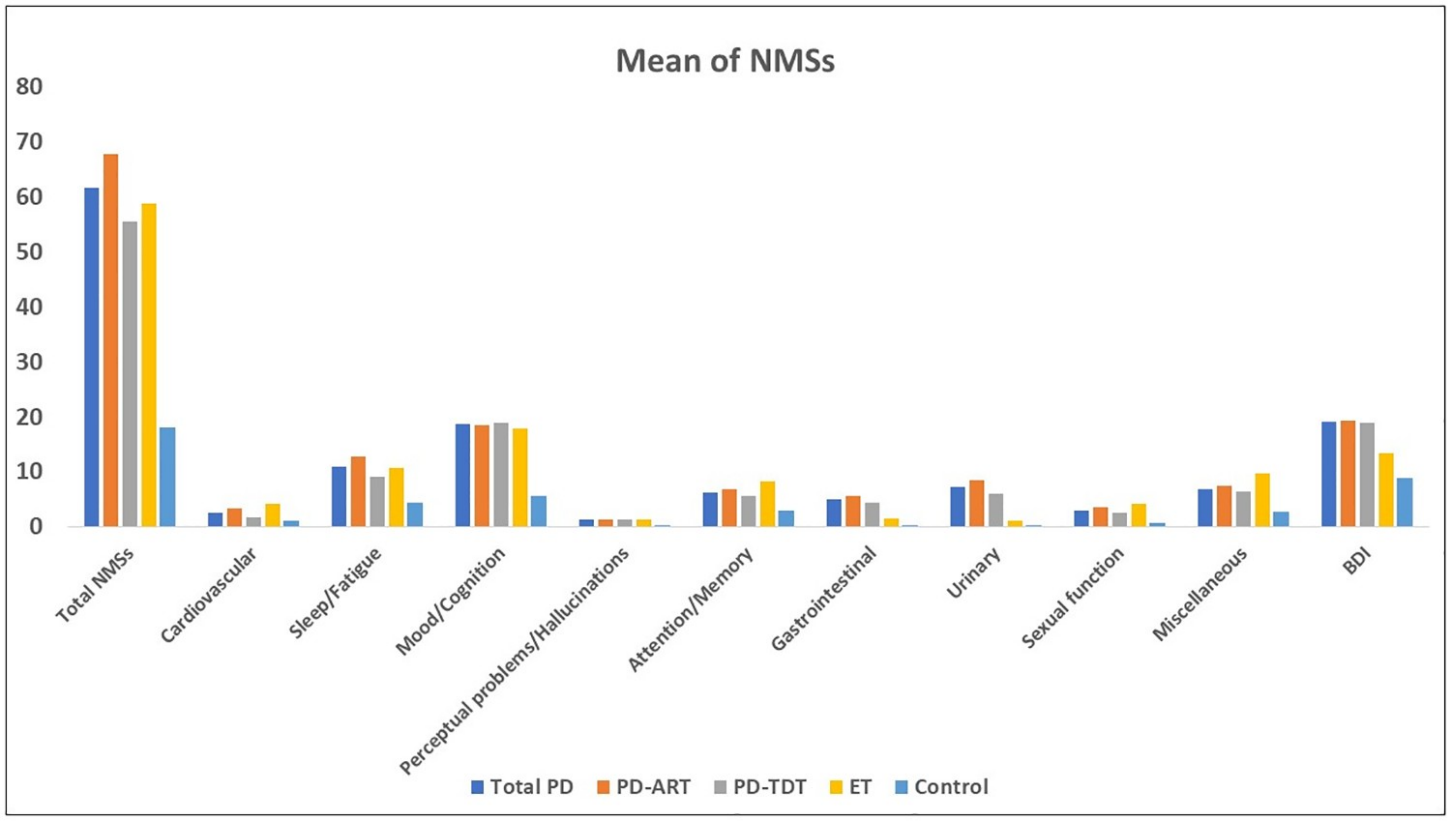
Means of NMSs in the study groups.

Table 2 shows the NMSs characteristics of different study groups. Patients with PD and ET had significant worse total NMSS (p<0.001), sleep/fatigue (p<0.001 and 0.002, respectively), mood/cognition (p<0.001 and 0.001, respectively), sexual (p = 0.047 and 0.011, respectively), and miscellaneous NMSs (p = 0.002 and <0.001, respectively), but not perceptual problems/ hallucinations domain (p = 0.154 and 0.222, respectively). However, Bonferroni correction showed that there was no significant difference between patients with PD and controls in terms of sexual symptoms (p = 0.054). Patients with PD, not ET, showed worse GIT and urinary symptoms compared to controls (p<0.001). Patients with ET, not PD, showed worse cerebrovascular symptoms (p = 0.005) and memory/ attention (p = 0.003), compared to controls. Compared to ET, patients with PD had significantly worse GIT (p = 0.002) and urinary symptoms (p = 0.001), while comparable severity of other domains (Fig 1).

**Table 2:** Non-motor characteristics of PD subtypes, ET and controls.

	Total PD Mean (SD)	PD-TDT	PD-ART	ET Mean (SD)	Control Mean (SD)
**Number**	72	33	33	29	28
**Total NMSs**	62.52 (40.64) *	55.54 (36.92) *	67.78 (44.65) *	58.72 (21.53) *	18.03 (11.67)
**Cardiovascular**	2.69 (4.02)	1.69 (2.36)	3.36 (5.03)	4.10 (3.63) *	1.07 (1.53)
**Sleep/fatigue**	11.27 (8.42) *	9.0 (6.45)	12.66 (8.35) *	10.75 (6.05) *	4.25 (3.55)
**Mood**	19.25 (15.89) *	18.84 (16.43) *	18.39 (15.97) *	17.89 (7.58) *	5.67 (3.74)
**Perceptual problems**	1.27 (3.05)	1.27 (3.59)	1.27 (2.73)	1.34 (1.89)	0.25 (0.64)
**Attention/memory**	5.81 (6.82)	5.57 (7.08)	6.81 (6.82)	8.17 (5.27) *	3.0 (3.12)
**Gastrointestinal**	4.88 (5.48) *†	4.27 (4.79) *	5.65 (6.31) *†	1.55 (2.73)	0.17 (0.47)
**Urinary**	7.21 (9.78) *†	6.06 (7.32) *	8.53 (12.27) *†	1.13 (1.92)	0.35 (0.73)
**Sexual function**	3.04 (5.68) *	2.42 (4.80)	3.54 (6.42)	4.13 (3.57) *	0.57 (1.28)
**Miscellaneous**	7.10 (6.83) *	6.39 (7.35)	7.43 (6.43) *	9.62 (4.94) *	2.67 (3.07)
**Depression sBDI**	18.80 (7.79) *†	18.97 (8.58) *†	19.29 (7.57) *†	13.44 (6.07)	8.92 (4.24)

PD, Parkinson’s Disease; ET, essential tremor; SD, standard deviation; TDT, tremor dominant type; ART, akinetic rigid type.

*P<0.05 compared to control.

^†^P<0.05 compared to ET group.

P-value was adjusted using Bonferroni method.

Using BDI, 84.7% of patients with PD had depression (mild, 44.3%; moderate, 29.2%; and severe,11.1%), while 69% of ET patients had depression (mild, 51.7%; moderate, 10.3%; and severe, 6.9%). Moreover, 28.6% of controls had depression (mild, 25%, and moderate, 3.6%). Patients with PD and ET patients had significantly worse BDI scores compared to controls (p<0.001 and 0.034, respectively), with worse scores among patients with PD than ET (p = 0.002). On the other hand, Bonferroni correction showed that BDI was comparable in both ET and control groups (p = 0.129).

Frequency of all NMS domains, except urinary symptoms was significantly more among ET patients compared to controls. PD patients showed more frequently impaired perception, GIT, urinary, sexual, and miscellaneous domains and depression, compared to controls. Compared to ET, PD patients showed significantly more frequently impaired GIT, urinary, and depression, and less frequently impaired memory, sexual and miscellaneous domains (Table 3). After the application of Bonferroni method, the difference between PD and ET was detected only in terms of urinary symptoms (p<0.001). Moreover, ET had higher frequency of sexual and miscellaneous symptoms compared to control group (p<0.001).

**Table 3:** Comparison of frequency of NMSs between different groups.

	PD-NMSs frequency	ET-NMS frequency	Controls-NMS frequency	PD vs ET	PD vs Control	ET vs Control	ART vs Control	TDT vs Control	TDT vs ET	ART vs ET	TDT vs ART
CVS	66.67%	79.31%	46.43%	0.208	0.062	0.01*	0.066	0.268	0.111	0.388	0.438
Sleep	94.45%	100%	82.14%	0.195	0.054	0.017*	0.053	0.15	0.178	0.345	0.555
Mood	88.89%	100%	85.71%	0.061	0.661	0.035*	0.282	0.682	0.016*	0.178	0.131
Perception	36.11%	44.82%	14.28%	0.416	0.032*	0.012*	0.029*	0.138	0.237	0.665	0.438
Memory/attention	72.22%	100%	75%	0.001*	0.721	0.004*	0.775	0.654	0.001*	0.007*	0.44
GIT	73.62%	44.82%	14.28%	0.008*	**<0.001**	0.012*	**<0.001**	**<0.001**	0.048*	0.007*	0.44
Urinary	76.40%	31.03%	21.43%	**<0.001**	**<0.001**	0.41	**<0.001**	**<0.001**	**<0.001**	0.001*	0.518
Sexual	43.05%	68.97%	17.86%	0.013*	0.027*	**<0.001**	0.149	0.012*	0.103	0.007*	0.248
Miscellaneous	79.16%	96.55%	57.14%	0.027*	0.032*	**<0.001**	0.02*	0.202	0.011*	0.111	0.253
BDI	92.24%	68.96%	28.57%	0.008*	**<0.001**	0.018*	**<0.001**	**<0.001**	0.039*	0.027*	0.786

PD, Parkinson’s Disease; ET, essential tremor; TDT, tremor dominant type; ART, akinetic rigid type.

*Non-significant after adjusting with Bonferroni correction.

### Comparison of non-motor symptoms of PD subtypes and ET groups

Compared to controls, PD-TDT and PD-ART groups had significant worse total NMS (p<0.001), mood/cognition (p = 0.001), GIT (p = 0.003 and <0.001, respectively), and urinary (p = 0.026 and <0.001, respectively). Miscellaneous NMS and sleep/fatigue symptoms were significantly worse in PD-ART, not PD-TDT, compared to controls (p = 0.016 and p<0.001, respectively).

Compared to ET, PD-TDT and PD-ART groups had comparable NMSs scores, except significantly worse GIT and urinary symptoms (p = 0.003 and 0.001, respectively) among PD-ART. Both PD-TDT and ART subtypes showed worse depression than the ET group (p = 0.018 and 0.009, respectively) and controls (p<0.001) (Fig 1).

Compared to ET, PD-ART and PD-TDT showed significantly more frequently impaired GIT, (p = 0.048 and 0.007, respectively) urinary, (p<0.001 and 0.001, respectively) and depression (p = 0.039 and 0.027, respectively), while less frequently impaired memory/attention (p = 0.001 and 0.007, respectively). Compared to PD-TDT, ET patients showed more frequently impaired miscellaneous domain. Compared to PD-TDT, PD-ART showed worse scores of depression, total NMSS and most of its domains, and more frequent NMSs except urinary and sexual domains, but without significant differences (Tables 2 and 3). Bonferroni method showed that there was no significant difference between both subtypes of PD in terms of NMSS frequency. Moreover, there was no significant difference between ET and PD-ART.

### Multivariate analysis of demographics, clinical characteristics, NMSs, and BDI in ET and PD

In the ET group, total NMSS were significantly associated to depression (β = 2.189, p<0.001), age of onset (β = 0.523, p = 0.032), and tremor severity (moderately) (r = 0.577, p = 0.047). Cardiovascular and memory/attention domains were significantly associated with BDI (β = 0.367, p = 0.001 and β = 0.422, p = 0.008, respectively) and disease severity (β = 0.156, p = 0.001 and β = 0.198, p = 0.004, respectively). Sleep/fatigue was associated with BDI (β = 0.471, p = 0.010), age (β = 0.170, p = 0.006), and age of onset (β = 0.170, p = 0.012). Furthermore, BDI was moderately associated with tremor severity (β = 0.166, p = 0.042) (Supplementary Table A in S1 File).

Regarding PD group, total NMSS and GIT domains were significantly associated with disease duration (β = 2.524, p = 0.045 and β = 0.409, p = 0.015, respectively), BDI (β = 2.869, p<0.001 and β = 0.29, p = <0.001, respectively), H&Y-Off (β = 11.389, p = 0.012 and β = 1.41, p = 0.023, respectively), and S&E-Off (β = -0.745, p<0.001 and β = -0.071, p = 0.016, respectively). Sleep/fatigue and memory domains were significantly associated with BDI (β = 0.472, p<0.001 and β = 0.338, p = 0.001, respectively), H&Y-Off (β = 3.122, p = 0.001 and β = 1.543, p = 0.046, respectively), and S&E-Off (β = -0.200, p<0.001 and β = -0.075, p = 0.045, respectively). Cardiovascular symptoms were associated with H&Y-Off (β = 1.023, p = 0.024, respectively) (Supplementary Table B in S1 File).

In terms of the PD-TDT subtype, total NMSs were significantly associated with BDI (β = 2.562, p<0.001). Cardiovascular and sleep domains were significantly associated with UPDRS II-Off (β = 0.083, p = 0.047 and β = 0.285, p = 0.008, respectively). Moreover, sleep/fatigue was significantly associated with BDI and S&E-Off (β = 0.475, p<0.001 and β = -0.136, p = 0.008, respectively) (Supplementary Table C in S1 File).

Regarding PD-ART, total NMSs was significantly associated with disease duration (β = 4.962, p = 0.006), UPDRS II-Off (β = 2.654, p = 0.015), UPDRS III-On (β = 1.227, p = 0.042), H&Y-Off (β = 17.879, p = 0.005), and S&E-Off (β = -1.213, p = 0.010). Sleep/fatigue and GIT domains were significantly associated with disease duration (β = 0.979, p = 0.003 and β = 0.753, p = 0.003, respectively), UPDRS II-Off (β = 0.357, p = 0.011 and β = 0.245, p = 0.016, respectively), BDI (β = 0.562, p = 0.001 and β = 0.485, p = 0.001, respectively), H&Y-Off (β = 3.613, p = 0.002 and β = 2.379, p = 0.008, respectively), and S&E-Off (β = -0.215, p = 0.001 and β = -0.128, p = 0.006, respectively). Mood/cognition and memory domains were associated with UPDRS II-Off (β = 0.716, p = 0.007 and β = 0.245, p = 0.016, respectively), BDI (β = 1.102, p = 0.001 and β = 0.485, p = 0.001, respectively), H&Y-Off (β = 6.256, p = 0.006 and β = 2.379, p = 0.008, respectively), and S&E-Off (β = -0.392, p = 0.001 and β = -0.128, p = 0.006, respectively) (Supplementary Table D in S1 File).

In PD group, BDI was significantly associated with disease duration of total PD (β = 0.761, p = 0.005), H&Y Off (β = 3.136, p<0.001), UPDRS II-Off (β = 0.431, p<0.001), and UPDRS III Off (β = 0.159, p = 0.001). In ET group, BDI was associated with disease severity (β = 0.166, p = 0.042).

## Discussion

Several previous studies confirmed that NMSs are an integral part of ET [20,21]; however, few studies compared NMS profiles of ET and PD motor subtypes [22,23]. The current study showed that both patients with ET and PD have worse several NMSs compared to controls and marked overlap with minimal significant differences between the two disorders. Patients with PD had significantly worse and more frequent gastrointestinal symptoms, urinary symptoms, and depression compared to ET. Compared to ET, similar differences were detected in PD-ART, while PD-TDT showed non-significant differences. To the best of our knowledge, this is the first study that compares both PD-TDT and PD-ART subtypes with ET in terms of NMSs.

Similar to current findings, Lee et al. reported a similar worse non-motor profile among patients with PD and ET compared to controls, with more frequent and worse gastrointestinal (by NMSS and SCOPA-AUT) and genitourinary symptoms (by SCOPA-AUT) among PD than ET and controls [6]. Similarly, Giorelli and his colleagues using the NMS questionnaire (NMS-Quest) showed a non-significant difference in the total number of NMSs between both ET and PD patients, with a slight increase among patients with PD [13]. However, they observed a significant increase in specific NMSs, e.g., drooling, orthostatic dizziness, and vivid dreaming in patients with PD. Different results could be explained by different assessment tools and different population characteristics, especially the older age of their groups and the low percentage of no-tremor patients (29%). Sengul et al. reported a similar frequency of most NMS-Quest items, with more frequently reported constipation, incomplete bowel emptying, and loss of taste /smell by patients with PD than ET patients [22].

Interestingly, autonomic dysfunctions, especially gastrointestinal and urinary symptoms, were more discriminative between both disorders, similar to prior studies [6,24]. Patients with PD had worse and more frequent symptoms compared to controls and ET, while ET patients were indifferent from controls. Consistently, Damian et al. reported worse autonomic scores of SCOPA-Aut of PD rather than ET patients [24]. In addition to more extensive brainstem neurodegeneration in PD, documented peripheral alpha-synuclein pathology in the spinal cord and enteric, parasympathetic nervous system in contrast to ET [24,25].

In the current study, depression was more reported and worse among patients with ET and PD than healthy controls. Moreover, patients with PD had more frequent and worse depression in relation to ET. Consistently, Lee et al. showed worse depression symptoms using the Montgomery-Asberg Depression Rating Scale in PD and ET patients, with higher scores among patients with PD [6]. Other previous studies showed similar depression scores between ET and PD patients [26–28]. Smeltere and his group demonstrated significantly more depression of both disorders than controls and more depression scores in patients with PD, but in contrast, no significant difference between PD and ET was detected [26].

Furthermore, another study by Miller et al. showed no significant difference between patients with ET, PD, and dystonia in terms of BDI, depression severity, or frequency [27]. A study by Puertas-Martın et al. compared 32 ET patients with long duration to 32 patients with PD with mild disease (stages I and II) with no significant difference [28]. These variable findings could be explained by the variability of assessment tools, the number of recruited patients, disease severity, disease duration, older age of patients, non-matched groups, and received medications. Remarkably, ET patients in the current study had worse FMTRS and long duration in relation to prior studies, which might explain the high non-motor burden. In contrast, most of our patients with PD were mild to moderate stages with a relatively shorter duration of prior studies that could affect the current findings.

On the other hand, the cardiovascular symptoms and memory/attention domains were remarkably impaired among ET patients in the current study. Similarly, Lee et al. detected the main affection of these domains in ET [6]. ET’s cognitive dysfunction was detected in the current study that is worse than controls and comparable to PD in consistent with previous studies [22,23,28]. Puertas-Martın et al. showed similar non-demented ET performance and mild PD in global cognitive functions, with marginal more worsening of memory and verbal fluency in ET [28]. Cognitive dysfunction in ET could be attributed to dysfunction of fronto-subcortical and cerebellar thalamocortical circuits [5,28].

In agreement with previous studies, correlations of NMSs were variable between ET and PD. In contrast to PD, ET NMSs did not correlate with disease duration and minimally correlated to tremor severity. Sengul et al. reported a correlation of NMS to disease severity among PD rather than ET patients [6,22]. Similarly, Lee et al. reported significant correlations of total NMSS and gastrointestinal scores with disease severity and duration among patients with PD compared to ET. They attributed different correlations to restricted and slow pathology of ET, specificity of NMSS to PD, and PD brainstem pathology [6]. Furthermore, It was observed that higher depression scores were associated with increased motor severity in both PD and ET patients, similar to previous studies [26,27].

Remarkably, PD-TDT showed higher scores of NMSs to ET, but non-significant, dissimilar to PD-ART. Furthermore, depression was more frequent and worse among PD-TDT. Moreover, PD-TDT showed significantly more frequently impaired GIT, urinary, and depression, while less frequently impaired memory/attention and miscellaneous domains compared to ET. Similarly, Kwon et al. showed similar severity of NMS of PD-TDT and ET, while PD-TDT patients had more frequent specific NMS symptoms, including hyposmia, RBD-like symptoms, urinary frequency, and memory impairment [23].

The aforementioned similarities between PD and ET in terms of total NMSs raise the question of whether there is an association between the two diseases. Several studies reported overlapping clinical motor features such as cogwheeling, postural instability, and rest tremor [29–32], and NMSs between both diseases [6,33,34]. In addition, many pathological changes were observed in the brains of patients with ET, such as a reduction of Purkinje cells (PCs) and a high incidence of torpedoes, PC axonal swellings [35,36]. These changes were accompanied by lower climbing fiber (CF) synaptic density and a higher percentage of CFs in parallel fiber territory [37], which have also been seen in patients with PD [38]. Symanski and colleagues compared PD and ET patients in terms of PC linear density, and they concluded that there was no significant difference [39]. In agreement, Rajput et al. failed to find any significant difference in the count of PCs between two groups of patients with PD and ET [40]. We suggest that this marked overlap could be explained by shared circuit dysfunction of both extrapyramidal disorders. Nevertheless, underlying mechanisms are different, with more degeneration in PD. Therefore, we believe that this overlap does not confirm a direct link and association between PD and ET.

Several studies investigated differences of NMSs between postural instability and gait disturbance (PIGD) and TDT motor subtypes of PD, aiming to characterize specific non-motor profiles and underlying pathophysiology [41]. Most studies documented worse NMSs among PIGD subtype [42]; however, few studies compared NMSs between PD-TDT and ART subtypes such as the current study. Compared to PD-TDT, our findings showed that the matched ART group had higher total NMSs, cardiovascular, sleep, fatigue, memory, gastrointestinal, urinary, and sexual symptoms, but without significant difference. Choi et al. reported no significant differences of BDI and NMSS between ART and TDT subtypes of de novo patients with PD [42]. Similarly, another study could not detect differences in non-motor burden between PD-ART and TDT [43]. Other studies showed more impairment of some NMS among patients with ART [44,45]. Wojtala et al. described less severe cognitive deficits in PD-TDT than PD-ART [46]. Variability of findings could be attributed to patients’ different clinical characteristics (disease duration, severity, and/or stage) between studies and compared groups. The lower burden of NMS in TDT is attributed to less advanced and diffuse dopaminergic and non-dopaminergic neurodegenerative changes than non-tremor dominant phenotypes, especially PIGD [41]. This could also explain the more burden of NMS of PD-ART compared to PD-TDT in relation to ET.

Non-tremor-dominant subtypes of PD have been demonstrated to have broader NMS features, more early autonomic features, and more cognitive disturbance in advanced stages. These characteristics are attributed to more complex and diffuse central and peripheral neurodegeneration and the involvement of diverse neurotransmitters, such as dopaminergic, serotonergic, cholinergic, and noradrenergic systems [41,47,48]. Additionally, NMSs fluctuations, especially mood swings, usually associate motor fluctuation-related to chronic dopaminergic use, which is mediated by oscillations of dopaminergic and non-dopaminergic mechanisms. Therefore, most NMSs are related to several underlying pathological and biochemical mechanisms [47,48]. Consequently, targeting different underlying mechanisms is essential for proper management of NMSs, including adjustment of dopaminergic medications and targeting other non-dopaminergic neurotransmitters.

Although the significant results of this study and the relatively large sample, there were some limitations, such as the small sample size of each group, the retrospective nature of this design introduces bias into the data collection, clinic-based study with an enrollment of patients with severe symptoms especially for ET, and the limited comprehensive assessment of cognitive functions and other NMSs. The current study’s strengths include a direct comparison of ET to ART and TDT PD subtypes, and matched demographics between ET and PD, and matched disease duration and severity between PD subtypes.

In conclusion, the current study described the disparity of non-motor profiles of ET and PD motor phenotypes and their correlates. ET patients had several nonmotor symptoms comparable to PD with more prominent differences of depression, gastrointestinal, and urinary symptoms between ET and PD-ART. Identifying these different profiles is important for predicting, better assessing, and tailoring the management of NMSs of ET and PD subtypes and implies the disparity of underlying pathophysiology and neurodegeneration.

## Supporting information

S1 File (DOCX)
