# Supplementary material for: Non-motor symptoms in essential tremor, akinetic rigid and tremor-dominant subtypes of Parkinson’s disease
Source: PLoS One. 2021 Jan 27;16(1):e0245918. doi: 10.1371/journal.pone.0245918 (PMC7840014; doi:10.1371/journal.pone.0245918)
Supplement: S1 File — (DOCX) [file pone.0245918.s001.docx]

Supplementary table A | Multivariate analysis of ET demographics, severity, BDI, and NMSs domains.

| ET | | BDI | Cardiovascular | Sleep and Fatigue | Mood and cognition | Perception and Hallucinations | Memory | GIT | Urinary | Sexual | Miscellaneous | NMS total | ET severity |
| --- | --- | --- | --- | --- | --- | --- | --- | --- | --- | --- | --- | --- | --- |
| Age | B | 0.003 | 0.017 | 0.170 | 0.068 | -0.022 | 0.077 | -0.023 | 0.016 | 0.048 | 0.019 | 0.37 | 0.129 |
|  | p-value | 0.963 | 0.668 | **0.006** | 0.416 | 0.283 | 0.179 | 0.448 | 0.454 | 0.215 | 0.726 | 0.11 | 0.395 |
| Disease Duration | B | -0.05 | -0.047 | 0.115 | -0.010 | -0.028 | -0.110 | -0.109 | -0.002 | -0.076 | -0.174 | -0.44 | -0.445 |
|  | p-value | 0.739 | 0.605 | 0.440 | 0.965 | 0.552 | 0.399 | 0.099 | 0.963 | 0.391 | 0.147 | 0.407 | 0.188 |
| Age of Onset | B | 0.015 | 0.030 | 0.170 | 0.079 | -0.019 | 0.112 | -0.001 | 0.019 | 0.072 | 0.062 | 0.523 | 0.239 |
|  | p-value | 0.831 | 0.486 | **0.012** | 0.374 | 0.384 | 0.064 | 0.974 | 0.401 | 0.077 | 0.286 | **0.032** | 0.135 |
| BDI | B |  | 0.367 | 0.471 | 0.701 | -0.034 | 0.422 | -0.019 | 0.062 | 0.082 | 0.136 | 2.189 | 0.865 |
|  | p-value |  | **0.001** | **0.010** | **0.002** | 0.570 | **0.008** | 0.832 | 0.307 | 0.468 | 0.385 | **0.001** | **0.042** |
| ET severity | B | 0.166 | 0.156 | 0.053 | 0.091 | 0.010 | 0.198 | -0.029 | -0.044 | 0.058 | 0.083 | 0.577 |  |
|  | p-value | **0.042** | **0.001** | 0.534 | 0.387 | 0.700 | **0.004** | 0.445 | 0.097 | 0.242 | 0.224 | **0.047** |  |

ET, essential tremor; BDI, Beck Depression Inventory; GIT, Gastrointestinal tract; NMS, Non-motor symptoms

Supplementary table B | Multivariate analysis of PD parameters, BDI, and NMSs domains.

| PD | | BDI | Cardiovascular | Sleep/ Fatigue | Mood and cognition | Perception and Hallucinations | Memory/attention | GIT | Urinary | Sexual | Miscellaneous | NMS total |
| --- | --- | --- | --- | --- | --- | --- | --- | --- | --- | --- | --- | --- |
| Age | B | -0.093 | -0.058 | 0.004 | -0.05 | -0.016 | 0.128 | 0.127 | 0.071 | 0.003 | 0.01 | 0.194 |
|  | p-value | 0.332 | 0.228 | 0.972 | 0.791 | 0.653 | 0.121 | 0.053 | 0.548 | 0.965 | 0.906 | 0.695 |
| Disease Duration | B | 0.761 | -0.03 | 0.675 | 0.831 | -0.045 | 0.116 | 0.409 | -0.124 | 0.146 | 0.553 | 2.524 |
|  | p-value | **0.005** | 0.808 | **0.008** | 0.088 | 0.631 | 0.589 | **0.015** | 0.685 | 0.434 | **0.008** | **0.045** |
| Age of Onset | B | -0.157 | -0.042 | -0.078 | -0.153 | -0.009 | 0.089 | 0.061 | 0.079 | -0.011 | -0.064 | -0.151 |
|  | p-value | 0.077 | 0.343 | 0.394 | 0.379 | 0.796 | 0.242 | 0.316 | 0.470 | 0.862 | 0.401 | 0.738 |
| UPDRS II  OFF | B | 0.431 | 0.124 | 0.397 | 0.473 | 0.051 | 0.089 | 0.137 | 0.091 | 0.034 | 0.266 | 1.678 |
|  | p-value | **<0.001** | **0.008** | **<0.001** | **0.010** | 0.16 | 0.272 | **0.033** | 0.435 | 0.617 | **0.001** | **<0.001** |
| UPDRS III OFF | B | 0.159 | 0.047 | 0.120 | 0.067 | 0.007 | 0.034 | 0.01 | -0.065 | -0.029 | 0.017 | 0.215 |
|  | p-value | **0.001** | 0.054 | **0.019** | 0.494 | 0.716 | 0.425 | 0.774 | 0.287 | 0.412 | 0.683 | 0.397 |
| H&Y Off | B | 3.136 | 1.023 | 3.122 | 3.314 | 0.245 | 1.543 | 1.41 | -0.344 | 0.33 | 0.731 | 11.389 |
|  | p-value | **<0.001** | **0.024** | **0.001** | 0.063 | 0.484 | **0.046** | **0.023** | 0.76 | 0.626 | 0.350 | **0.012** |
| S&E  OFF | B | -0.194 | -0.057 | -0.200 | -0.252 | 0.003 | -0.075 | -0.071 | -0.009 | -0.008 | -0.075 | -0.745 |
|  | p-value | **<0.001** | **0.009** | **<0.001** | **0.003** | 0.866 | **0.045** | **0.016** | 0.872 | 0.809 | **0.043** | **<0.001** |
| BDI | B |  | 0.072 | 0.472 | 1.016 | 0.026 | 0.338 | 0.29 | 0.176 | 0.174 | 0.343 | 2.869 |
|  | p-value |  | 0.268 | **<0.001** | **<0.001** | 0.604 | **0.001** | **<0.001** | 0.267 | 0.078 | **0.001** | **<0.001** |

PD, Parkinson’s Disease; H&Y, Hoehn and Yahr Scale; S&E, Schwab and England Scale, UPDRS, Unified Parkinson’s Disease Rating Scale; BDI, Beck Depression Inventory; GIT, Gastrointestinal tract; NMS, Non-motor symptoms

Supplementary table C | Multivariate analysis of PD-TDT parameters, BDI, and NMSs domains.

| PD TDT | | BDI | Cardiovascular | Sleep and Fatigue | Mood and Cognition | Perception and Hallucinations | Memory | GIT | Urinary | Sexual | Miscellaneous | NMS total |
| --- | --- | --- | --- | --- | --- | --- | --- | --- | --- | --- | --- | --- |
| Age | B | -0.041 | 0.015 | -0.056 | -0.316 | -0.022 | 0.096 | 0.138 | 0.86 | 0.078 | 0.038 | 0.058 |
|  | P-value | 0.826 | 0.751 | 0.675 | 0.344 | 0.761 | 0.51 | 0.156 | 0.565 | 0.426 | 0.801 | 0.939 |
| Disease Duration | B | 0.843 | 0.166 | 0.293 | -0.571 | -0.318 | 0.073 | 0.018 | -0.283 | 0.054 | 0.164 | -0.402 |
|  | P-value | 0.068 | 0.183 | 0.392 | 0.512 | 0.09 | 0.846 | 0.945 | 0.468 | 0.832 | 0.675 | 0.838 |
| Age of Onset | B | -0.135 | 0.003 | -0.073 | -0.211 | 0.019 | 0.061 | 0.13 | 0.115 | 0.066 | 0.004 | 0.114 |
|  | P-value | 0.421 | 0.940 | 0.543 | 0.490 | 0.774 | 0.648 | 0.142 | 0.399 | 0.459 | 0.976 | 0.869 |
| UPDRS II  Off | B | 0.46 | 0.083 | 0.285 | 0.142 | -0.003 | 0.076 | 0.012 | -0.038 | 0.01 | 0.168 | 0.735 |
|  | P-value | **0.001** | **0.047** | **0.008** | 0.626 | 0.966 | 0.555 | 0.886 | 0.779 | 0.91 | 0.204 | 0.267 |
| UPDRS III  Off | B | 0.156 | 0.031 | 0.103 | 0.010 | 0.001 | 0.021 | -0.034 | -0.011 | -0.023 | -0.026 | 0.071 |
|  | P-value | **0.040** | 0.121 | 0.057 | 0.945 | 0.982 | 0.725 | 0.414 | 0.859 | 0.58 | 0.678 | 0.822 |
| H&Y  Off | B | 2.947 | 0.383 | 1.027 | -1.781 | -0.779 | 1.776 | -0.346 | -0.877 | -0.308 | -1.233 | -2.138 |
|  | P-value | 0.059 | 0.4 | 0.401 | 0.568 | 0.261 | 0.189 | 0.71 | 0.534 | 0.741 | 0.384 | 0.759 |
| S&E  Off | B | -0.189 | -0.012 | -0.136 | -0.097 | 0.060 | -0.075 | 0.004 | 0.064 | 0.025 | -0.027 | -0.194 |
|  | P-value | **0.004** | 0.556 | **0.008** | 0.482 | **0.047** | 0.208 | 0.915 | 0.300 | 0.542 | 0.666 | 0.528 |
| BDI | B |  | 0.1 | 0.475 | 1.053 | 0.001 | 0.346 | 0.138 | 0.04 | 0.089 | 0.321 | 2.562 |
|  | P-value |  | 0.056 | **<0.001** | **0.001** | 0.997 | **0.024** | 0.198 | 0.811 | 0.421 | **0.043** | **<0.001** |

TDT, tremor dominant type; H&Y, Hoehn and Yahr Scale; S&E, Schwab and England Scale, UPDRS, Unified Parkinson’s Disease Rating Scale; BDI, Beck Depression Inventory; GIT, Gastrointestinal tract; NMS, Non-motor symptoms

Supplementary table D| Multivariate analysis of PD-ART parameters, BDI, and NMSs domains.

| PD-ART | | BDI | Cardiovascular | Sleep/ Fatigue | Mood and cognition | Perception and Hallucinations | Memory | GIT | Urinary | Sexual | Miscellaneous | NMS total |
| --- | --- | --- | --- | --- | --- | --- | --- | --- | --- | --- | --- | --- |
| Age | B | -0.115 | -0.092 | 0.115 | 0.205 | -0.015 | 0.174 | 0.142 | 0.127 | -0.083 | 0.027 | 0.582 |
|  | P-value | 0.347 | 0.251 | 0.387 | 0.422 | 0.752 | 0.109 | 0.16 | 0.523 | 0.427 | 0.795 | 0.421 |
| Disease  Duration | B | 1.105 | -0.031 | 0.979 | 1.955 | 0.126 | 0.213 | 0.753 | 0.032 | 0.186 | 0.847 | 4.962 |
|  | P-value | **0.006** | 0.883 | **0.003** | **0.002** | 0.271 | 0.462 | **0.003** | 0.951 | 0.543 | **0.001** | **0.006** |
| Age of Onset | B | -0.184 | -0.075 | -0.02 | -0.063 | -0.029 | 0.12 | 0.027 | 0.019 | -0.086 | -0.079 | -0.117 |
|  | P-value | 0.107 | 0.31 | 0.87 | 0.788 | 0.477 | 0.234 | 0.775 | 0.401 | 0.365 | 0.405 | 0.861 |
| UPDRS II  Off | B | 0.524 | 0.15 | 0.357 | 0.716 | 0.107 | 0.155 | 0.245 | 0.211 | -0.121 | 0.341 | 2.367 |
|  | P-value | **0.001** | 0.086 | **0.011** | **0.007** | **0.021** | 0.187 | **0.016** | 0.323 | 0.485 | **0.001** | **0.001** |
| UPDRS III  Off | B | 0.183 | 0.073 | 0.087 | 0.104 | 0.012 | 0.06 | 0.056 | -0.132 | 0.098 | 0.058 | 0.302 |
|  | P-value | **0.014** | 0.128 | 0.28 | 0.501 | 0.641 | 0.367 | 0.361 | 0.269 | 0.272 | 0.352 | 0.488 |
| H&Y  Off | B | 3.737 | 1.116 | 3.613 | 6.256 | 0.883 | 1.6 | 2.379 | -0.344 | 0.701 | 1.889 | 17.879 |
|  | P-value | **0.001** | 0.134 | **0.002** | **0.006** | **0.025** | 0.112 | **0.008** | 0.852 | 0.490 | **0.044** | **0.005** |
| S&E  Off | B | -0.222 | -0.081 | -0.215 | -0.392 | -0.044 | -0.076 | -0.128 | -0.044 | -0.035 | -0.11 | -1.116 |
|  | P-value | **0.001** | **0.032** | **0.001** | **0.001** | **0.031** | 0.149 | **0.006** | 0.649 | 0.491 | **0.022** | **0.001** |
| BDI | B |  | 0.051 | 0.562 | 1.102 | 0.058 | 0.291 | 0.485 | 0.326 | 0.36 | 0.418 | 3.514 |
|  | P-value |  | 0.684 | **0.001** | **0.001** | 0.378 | 0.051 | **0.001** | 0.284 | **0.049** | **0.004** | **0.001** |

ART, akinetic rigid type; H&Y, Hoehn and Yahr Scale; S&E, Schwab and England Scale, UPDRS, Unified Parkinson’s Disease Rating Scale; BDI, Beck Depression Inventory; GIT, Gastrointestinal tract; NMS, Non-motor symptoms
